# Beyond bacteria: large agents with analogies to Mimiviruses detected in canine cancers: reexamining Gram staining in cancer diagnostics

**DOI:** 10.3389/fvets.2026.1759298

**Published:** 2026-02-20

**Authors:** Elena Angela Lusi, Federico Caicci, Viola Zappone, Marco Quartuccio, Ilaria Dragà, Antonio Ieni, Cornelia Mannarino, Giuseppe Mazzullo, Claudia Rifici

**Affiliations:** 1 St Vincent Health Care Group-UCD, Dublin, Ireland; 2 Department of Biology, Electron Microscopy Unit, University of Padua, Padua, Italy; 3 Department of Veterinary Sciences, University of Messina, Messina, Italy; 4 Department of Human Pathology in Adult and Developmental Age "Gaetano Barresi", Section of Pathology, University of Messina, Messina, Italy

**Keywords:** canine cancer, Gram staining, human cancer, Mimivirus-like agent, oncogenic agent

## Abstract

While Gram staining is traditionally used for classifying bacteria based on their cell wall properties, Mimiviruses and large mammalian agents can also retain the Gram stain, despite not being typical bacteria. In fact, Mimivirus-like agents that exhibit Gram-positive staining were first found in human tissues, particularly in malignant samples, suggesting that these agents may be involved in a unique carcinogenic process. In order to translate the findings published in human medicine to animal models, we evaluated for the first time the presence of analogous Gram-positive agents in canine malignancies and differentiate them from traditional bacteria. Using Gram staining, we analyzed 35 canine tumors across various malignancy types, including 7 sarcomas, 15 carcinomas, and 13 round cell tumors such as mast cell tumors, transmissible venereal tumors and melanomas. Normal tissues and bacteria were used as controls. We were able to identify Gram-positive granulations, exhibiting intracytoplasmic, intra-nuclear and perinuclear patterns, measuring 1–2 μm that were distinct from traditional bacteria. This study, the first of its kind in the veterinary literature, supports comparable published findings in human research and advances our knowledge of the pathophysiology of cancer across species.

## 1 Introduction

The discovery of Mimiviruses, large DNA viruses that infect amoebas, challenged traditional distinctions between viruses and cellular organisms and prompted significant reevaluation in microbiology and virology. In particular, their discovery has challenged existing paradigms regarding the complexity and classification of viruses, introduced the concept that viruses can be much larger than previously though and blurred lines between viral and cellular life forms (1–9). The hybrid nature of Mimivirus is enclosed in their name “Mimiviruses” that etymologically means “mimicking microbes” (10). Despite being viruses, they exhibit bacterial characteristics like retaining Gram stain, which is typically used to color bacteria (11–15).

Subsequently, recent studies have identified large mammalian agents in human tissues, notably in cancerous ones, which resemble Mimiviruses due to their Gram stain retention and considerable size. However, unlike Mimiviruses, these agents differ markedly in their biological nature and tissue tropism, as they can infect mammalian cells directly without relying on amoeba hosts and exhibit transforming capabilities, implying a possible role in cancer development and emphasizing their significance in oncological research (16–18).

We were eager to apply these findings to animal cancer because of the topic’s novelty, the dearth of published literature on the subject and the lack of comparable findings in veterinary oncology.

The current study extends these observations to animal models by applying Gram staining to formalin-fixed tissues of canine cancer specimens, enabling the identification of large, previously unrecognized agents within tumors. We present detailed morphological comparisons of these granules with conventional bacteria and highlight conceptual analogies with Mimiviruses, while emphasizing that these mammalian entities differ substantially in biological organization and tissue tropism. These findings suggest a potential novel role for such agents in oncogenesis and underscore the need for further research to clarify their biological significance and possible implications in both veterinary and comparative oncology.

## 2 Materials and methods

The study included 35 canine tumors, categorized into sarcomas (7 cases), carcinomas (15 cases), and round cell tumors (13 cases). all 35 canine tumor specimens were diagnosed prior to Gram staining using standard veterinary histopathological criteria, including hematoxylin–eosin staining and routine pathological evaluation by board-certified veterinary pathologists. Detailed information on tumor type, number of cases, and representative histological subtypes is summarized in Table 1.

**Table 1:** Summary of canine tumor samples analyzed by Gram staining.

Tumor type	Number of cases	Histological subtypes (examples)	Gram-positive granules observed	Notes
Sarcomas	7	Fibrosarcoma, Osteosarcoma, Hemangiosarcoma, Liposarcoma	Yes, intracytoplasmic & perinuclear	Granules prominent in undifferentiated regions
Carcinomas	15	Mammary carcinoma, Hepatoid gland carcinoma, Adenocarcinoma, Squamous cell carcinoma	Yes, intracytoplasmic & perinuclear	Granules concentrated near aggressive tumor areas
Round cell tumors	13	Mast cell tumor, Transmissible venereal tumor, Melanoma	Yes, cytoplasmic & punctate	Morphologically distinct from typical granules/pigments

The table presents the distribution of canine tumors examined in this study, including sarcomas, carcinomas and round cell tumors. For each tumor type, the number of cases, representative histological subtypes, presence of Gram-positive granules and key observations are summarized. Observations are descriptive; no statistical inference was applied.

### 2.1 Gram staining on fixed tissues

The staining process was carried out in accordance with Lusi et al. (16)’s protocol which adapted the Gram stain technique for application to tissue samples, involving a series of hydration steps, microwave heating, and sequential application of crystal violet, iodine, decolorizer, and safranin, followed by dehydration and mounting for microscopic examination, enabling the differentiation of Gram-positive granules within fixed animal cancer tissues (the characteristics of animals samples are detailed in Table 1). The tissue blocks fixed in formalin and embedded in paraffin were retrieved from the archives of the Veterinary Pathology Unit of the Department of Veterinary Sciences at the University of Messina. For each block, 5 μm sections were cut, placed on poly-l-lysine-coated slides, and stained using the Gram technique. Briefly, the slides were hydrated by immersing them for 5 min in xylene I, for 5 min in xylene II, and subsequently in a decreasing alcohol series, from absolute alcohol to 90, 80, 70, 60, 50%, until reaching distilled water. The slides were placed in a plastic tray containing distilled water, then heated in a microwave at 850 W for 5 min, before performing the Gram stain. Gram stain was performed in accordance with the manufacturer’s instructions. Observations were made using an optical microscope (DMI6000, Leica) equipped with a camera and image processing software for detailed analysis. Purified large agents obtained via sucrose gradient fractionation were also Gram-stained to confirm that the intracytoplasmic, perinuclear, and occasional intranuclear granules observed in tissue corresponded to distinct, isolated biological entities.

### 2.2 Purification of the large agents from cancer specimens and electron microscopy

The isolation procedure involved homogenizing canine cancer specimens, after surgical excision, with PBS and protease inhibitors at 4 °C to obtain a crude extract, followed by centrifugation to remove debris. The supernatant was then layered onto a 25% sucrose cushion for ultracentrifugation for 2 h to concentrate large particles, resulting in a white fraction that was further purified by an additional 30-min centrifugation cycle to precipitate the particles. For electron microscopy 25 μL of the purified fraction was placed on a Holey Carbon grid, stained with uranyl acetate, and observed under a Tecnai G2 transmission electron microscope at 100 kV, with images captured digitally.

## 3 Results

**Figure 1:**
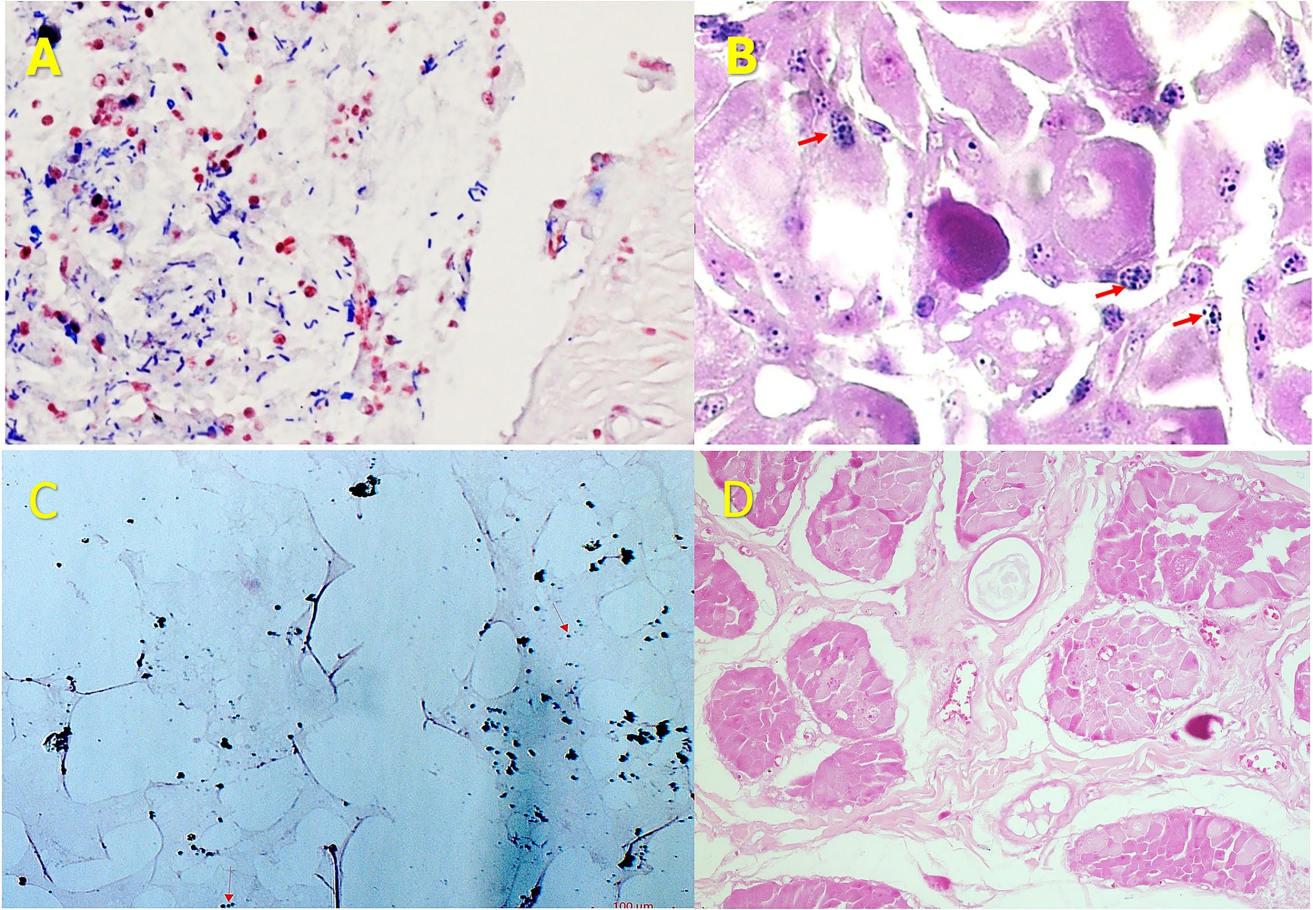
**(A)** Gram-positive bacterial cocci used as control. **(B)** Canine hepatoid gland cancer with fine Gram-positive granules. The elongated or rod-shaped cocci in image **(A)** contrast with the fine, punctate granules in image **(B)**, which are dispersed throughout the cytoplasm and surrounding the nucleus, indicating distinct morphological features. Section B uses red arrows to highlight blue granules, helping readers learn to recognize them so they can identify these particles on their own in later sections without arrows. **(C)** Purified particles after sucrose-gradient fractionation, retaining Gram positivity. **(D)** Normal hepatoid gland tissue processed with Gram stain, showing absence of these granules.

We identified Gram-positive granules within tumor tissues, predominantly located in the cytoplasm and perinuclear regions, with occasional nuclear localization, across various histopathology types, including sarcomas and carcinomas. These bluish granules, measuring 1–2 μm, were morphologically distinct from classical bacteria used as controls and were particularly prominent in undifferentiated neoplastic regions. Purified agents obtained through sucrose gradient fractionation were also Gram-stained, confirming that the granules observed in tissue sections corresponded to discrete, isolated biological entities rather than nonspecific staining artifacts. Negative control slides from normal, non-neoplastic tissues showed no detectable Gram-positive granules under identical staining conditions (Figure 1).

**Figure 2:**
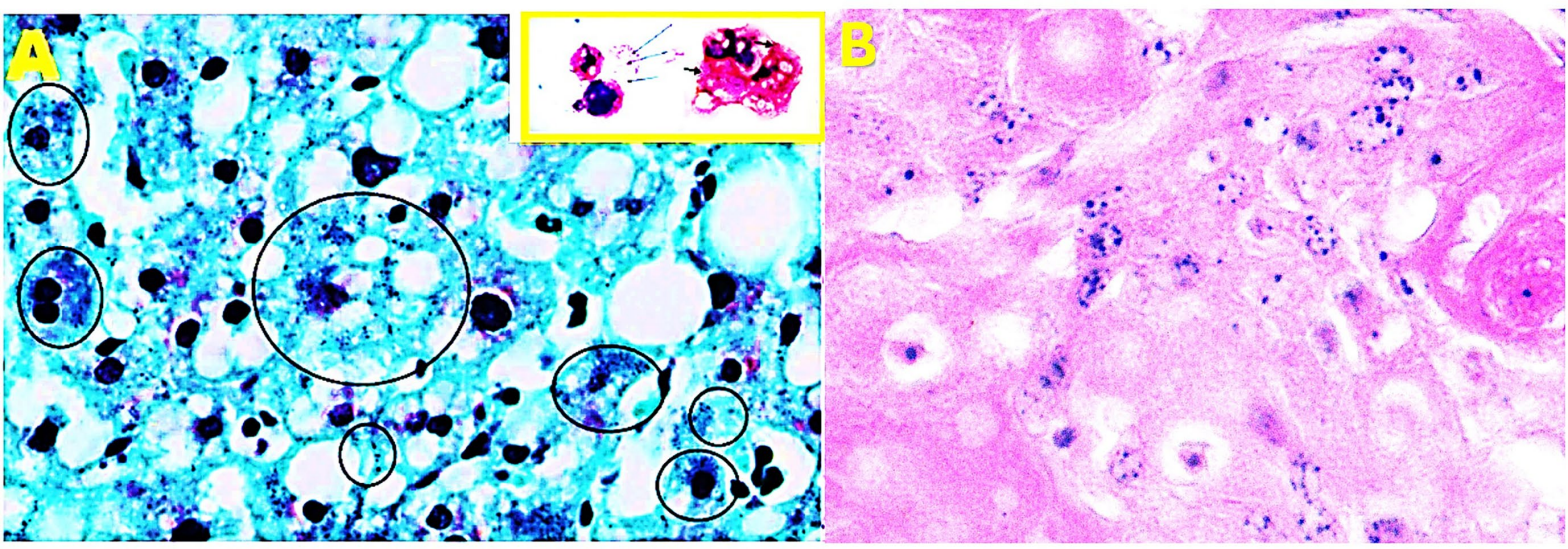
**(A)** Gram staining of a human liver (magnification, ×80). After the Gram stain, human liver cells displayed fine blue granules that, for didactic reasons, are enclosed in the black circles, but they can be seen scattered in the parenchyma. Note the similarities between the amoebal Mimiviruses appearing as blue granules (small picture yellow frame) with the human blue granules. In the human cells, the gram positive granules appear as fine granules and *are distinct* from bacteria and other pigment, like lipofucsin, that is also present (brown color). **(B)** Hepatoid gland carcioma, note the same fine blue granules.

Notably, in hepatoid gland carcinomas, these granules appeared clear and uniform, resembling those previously described in human tissues (Figure 2). In breast tumors, large clusters of Gram-positive structures were observed in stromal areas adjacent to highly aggressive tumor regions, suggesting potential interactions with the tumor microenvironment.

**Figure 3:**
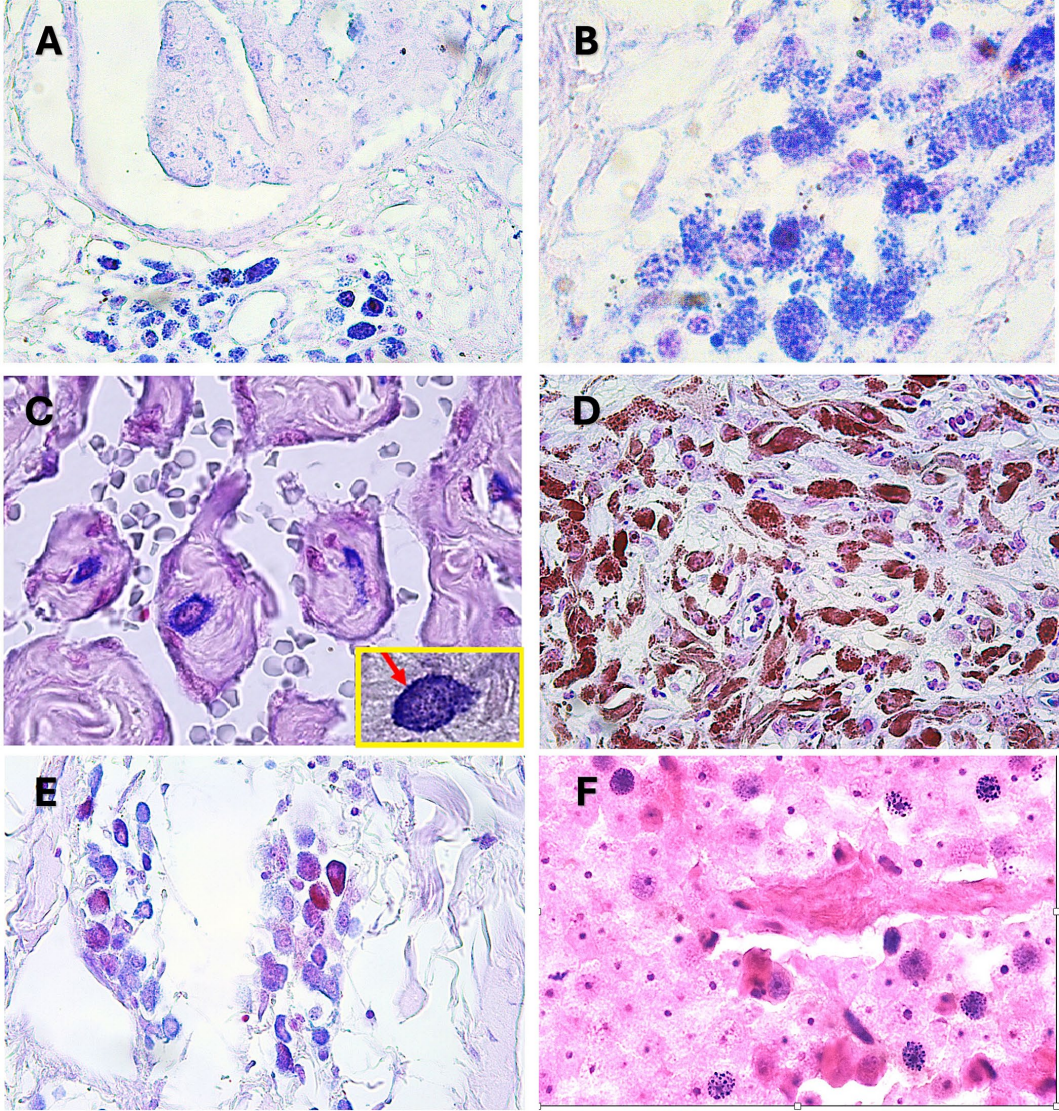
Representative sections of canine cancer specimens stained with Gram: **(A)** Mixed breast cancer with groups of positive cells, 40×; **(B)** Detail of the previous image, showing small bluish granulations occupying the entire cytoplasm of the cells, 100×; **(C)** Undifferentiated canine sarcoma with blue granules in mesenchimal cells, 40×; in the yellow frame a sarcoma cancer cell with an oval appearance strongly positive (red arrow), 100×; **(D)** Canine melanoma showing the presence of fine punctuate gram positive (blue) granules that are distinct from melanine brown granules; **(E)** Canine mastocytoma that shows the difference between the typical mast cell granules and the Gram-positive structures inside tumor cells, 40×; **(F)** Transmissible venereal tumor (TVT) with dispersed positive cells displaying pinpoint positive granules, 40×.

**Figure 4:**
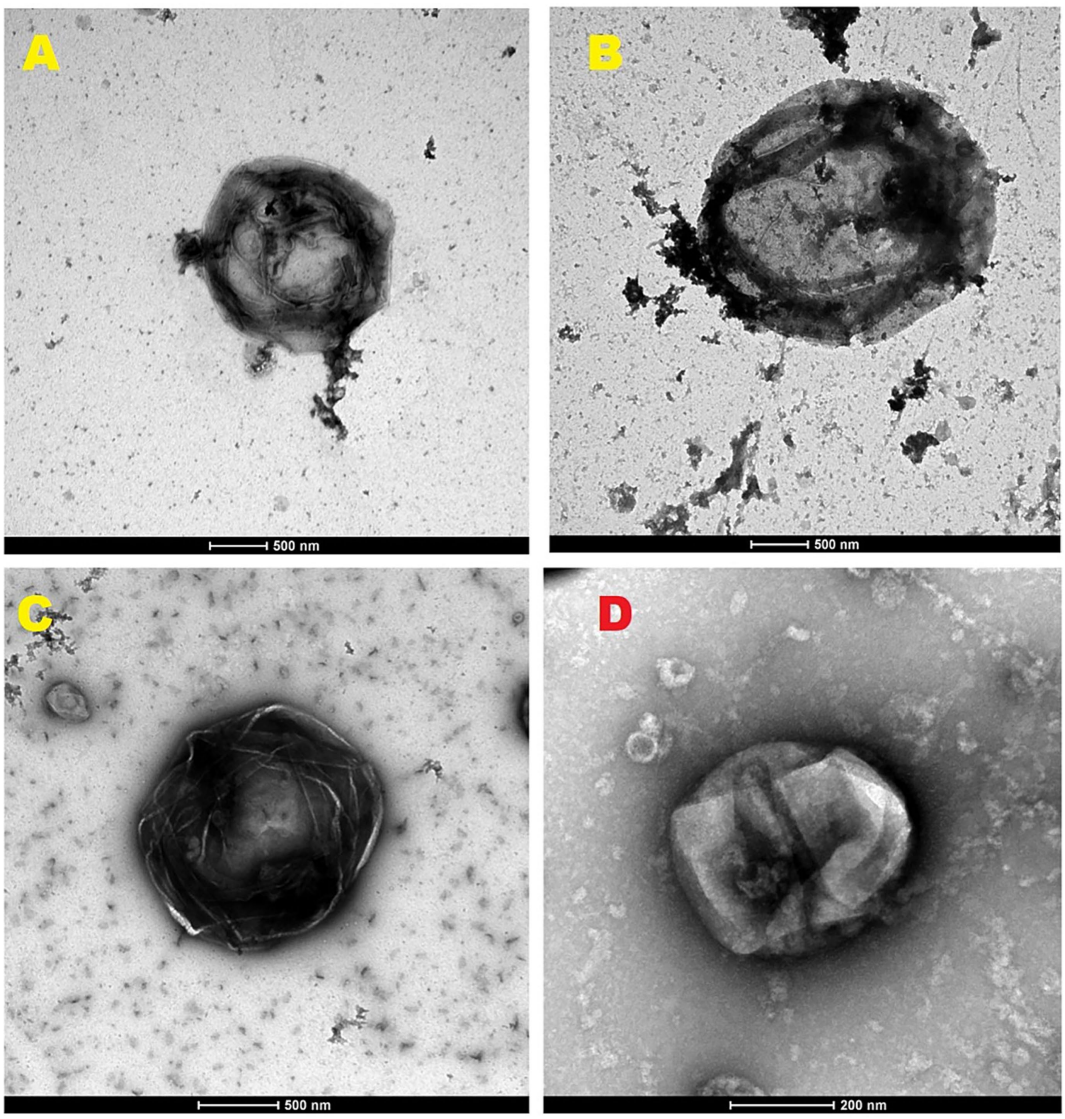
Electron microscopy of the large mammalian agents (1–3 micron in size) that retain the Gram staining, after isolation and purification through a sucrose gradient. **(A–C)** Agents isolated from human cancer cells. **(D)** Agent isolated from canine breast CA.

The presence of Gram-positive granules—both cytoplasmic and perinuclear—was consistent across multiple canine tumor types, including sarcomas, carcinomas, hepatoid gland tumors, melanomas, and round cell tumors such as mastocytoma and transmissible venereal tumor (Figure 3). These granules were particularly concentrated in stromal regions adjacent to aggressive tumor zones, suggesting a possible role in tumor progression. Figure 4 illustrates the electron microscopy images of the gram-positive granules, which are large agents measuring 1–2 microns, similar in size and gram stain retention to Mimiviruses, but distinct in their biological properties. Unlike Mimiviruses, these granules do not require amoeba coculture for isolation and appear to have a different tropism, suggesting they may represent a novel class of large infectious agents with unique host interactions.

## 4 Discussion

The discovery of Mimiviruses—giant DNA viruses capable of retaining Gram stain despite being viruses—has profoundly challenged traditional boundaries between viruses and cellular organisms and prompted a re-examination of long-standing microbiological assumptions (1–9). Building on this paradigm shift, Lusi et al. reported the unexpected presence of Gram-positive blue granules in human tissues, particularly within malignant samples, noting striking morphological similarities to Mimiviruses previously described in amoebae (16). Importantly, those observations were not intended to imply viral identity, but rather to highlight the existence of unusually large biological entities detectable by a classical bacteriological stain.

Starting from these premises, the present study aimed to determine whether analogous Gram-positive entities could be reproducibly detected in animal tumors, with a particular focus on canine neoplasms. Dogs represent a well-established and valuable model in comparative and translational oncology, as they develop spontaneous tumors in a shared environment with humans and exhibit strong morphological, biological, and diagnostic similarities to human cancers (19–22). Unlike murine models, which frequently rely on artificial tumor induction, canine cancers offer a naturally occurring context that enhances translational relevance.

By applying an adapted Gram-staining protocol exclusively to previously diagnosed canine tumors, we identified consistent Gram-positive granules within neoplastic tissues across multiple histological types. These entities displayed intracytoplasmic, perinuclear, occasionally intranuclear localization patterns and measured approximately 1–2 μm in diameter. Their morphology and staining behavior were clearly distinct from classical bacteria used as controls, as well as from endogenous cellular pigments. These findings parallel those previously reported in human cancer tissues and support the reproducibility of this staining-based observation across species.

To strengthen the methodological coherence of these observations, the analysis was extended beyond tissue sections by purifying the same entities through sucrose-gradient fractionation. Notably, the purified particles retained Gram positivity when stained independently and exhibited comparable size and morphology under electron microscopy. This experimental continuity confirms that the Gram-positive granules observed *in situ* correspond to biological entities rather than nonspecific staining artifacts or dye precipitates, reinforcing the technical validity of the protocol presented. Importantly, this work demonstrates that Gram staining of fixed tissue sections can serve as a reliable methodological tool for detecting these previously unrecognized agents, and that technical challenges such as slide preparation and staining can be systematically overcome—for example, through careful preheating of slides prior to staining.

The coexistence of these Gram-positive entities with neoplastic tissue raises broader questions regarding the complex relationship between infection and carcinogenesis. From both veterinary and human health perspectives, infectious oncogenesis represents a multifaceted and clinically relevant field in which causality, opportunism, and coexistence often overlap. In this context, the absence of detectable Gram-positive entities in normal tissues processed under the same experimental conditions should be interpreted cautiously. Rather than representing definitive absence, this finding constitutes an observational outcome of the conditions employed. Analogous to cellular oncogenes—which are intrinsically present in normal cells but acquire pathological relevance only upon activation—it remains conceivable that similar entities may exist in non-neoplastic tissues at undetectable levels or in inactive states, becoming apparent only during or after malignant transformation.

While functional studies—including tumorigenic assays in cell-based systems and animal models (mice and dogs)—have been conducted in parallel research, with some results already published and others forthcoming, these findings lie outside the scope of the present methodological report and are therefore not discussed here. Likewise, molecular and genomic characterization of these entities is ongoing or completed in separate investigations and will be reported in dedicated publications. The primary aim of this manuscript is methodological: to present a reproducible staining approach capable of revealing previously unrecognized large entities within animal tumors. In this context, Gram staining is used strictly as a descriptive and exploratory screening tool, rather than as a method for taxonomic or etiological identification. The term “Mimivirus-like” is employed solely in a comparative, morphological sense to facilitate conceptual understanding since these mammalian entities share certain analogies with Mimiviruses—particularly their large size and ability to retain Gram stain—but they differ substantially from known Mimiviruses in biological organization, tissue tropism, and host interaction.

Taken together, these findings support a dual oncological–infectious paradigm in which neoplastic and potentially infectious components coexist within the tumor microenvironment, reflecting a biological continuum rather than distinct, isolated mutually exclusive processes. Within the broader context of comparative oncology—and in line with current efforts to critically reassess what is known, what remains uncertain and what may have been overlooked regarding infectious agents in animal cancers (23) this work provides a methodological foundation for future functional, molecular, and translational investigations aimed at clarifying the biological nature, origin, and possible oncogenic significance of these novel large agents across species.

## 5 Conclusion

This study demonstrates that a simple Gram stain can serve as an effective initial screening tool to detect large, previously unrecognized agents in both human and canine cancers. The presence of Mimivirus-like agents exhibiting Gram-positive staining and retroviral antigenicity suggests a novel class of oncogenic agents with unique biological properties. Observations support a dual oncological–infectious framework, highlighting the coexistence of neoplastic and potentially infectious components, with implications for diagnostics, prognosis and future research. While descriptive in nature, these findings underscore the translational value of comparative oncology and encourage further studies to elucidate the biological significance and therapeutic potential of these agents.

## Data Availability

The original contributions presented in the study are included in the article/supplementary material, further inquiries can be directed to the corresponding author.
